# Inertial measurement systems for segments and joints kinematics assessment: towards an understanding of the variations in sensors accuracy

**DOI:** 10.1186/s12938-017-0347-6

**Published:** 2017-05-15

**Authors:** Karina Lebel, Patrick Boissy, Hung Nguyen, Christian Duval

**Affiliations:** 1 Faculty of Medicine and Health Sciences, Orthopedic service, Department of Surgery, Université de Sherbrooke, Sherbrooke, QC J1H 5N4 Canada; 2 Research Center on Aging, 1036 Belvédère Sud, Sherbrooke, QC J1H 4C4 Canada; 3 Interdisciplinary Institute for Technological Innovation (3IT), Université de Sherbrooke, Sherbrooke, QC J1K 0A5 Canada; 4 Département des Sciences de l’activité Physique, Université du Québec à Montréal, Montreal, QC H2X 1Y4 Canada; 5 Centre de Recherche Institut Universitaire de Gériatrie de Montréal, Montreal, QC H3W 1W4 Canada

**Keywords:** AHRS, IMU, Inertial sensors, Attitude and heading reference system, 3D orientation tracking, Inertial motion capture, Sit-to-stand, Gait, Walk, Turn, Validation, Accuracy

## Abstract

**Background:**

Joints kinematics assessment based on inertial measurement systems, which include attitude and heading reference system (AHRS), are quickly gaining in popularity for research and clinical applications. The variety of the tasks and contexts they are used in require a deep understanding of the AHRS accuracy for optimal data interpretation. However, published accuracy studies on AHRS are mostly limited to a single task measured on a limited number of segments and participants. This study assessed AHRS sensors kinematics accuracy at multiple segments and joints through a variety of tasks not only to characterize the system’s accuracy in these specific conditions, but also to extrapolate the accuracy results to a broader range of conditions using the characteristics of the movements (i.e. velocity and type of motion). Twenty asymptomatic adults ($\overline{age}$ = 49.9) performed multiple 5 m timed up and go. Participants’ head, upper trunk, pelvis, thigh, shank and foot were simultaneously tracked using AHRS and an optical motion capture system (gold standard). Each trial was segmented into basic tasks (sit-to-stand, walk, turn).

**Results:**

At segment level, results revealed a mean root-mean-squared-difference $\overline{(RMSD)}$ varying between 1.1° and 5.5° according to the segment tracked and the task performed, with a good to excellent agreement between the systems. Relative sensor kinematics accuracy (i.e. joint) varied between 1.6° and 13.6° over the same tasks. On a global scheme, analysis of the effect of velocity on sensor kinematics accuracy showed that AHRS are better adapted to motions performed between 50°/s and 75°/s (roughly thigh and shank while walking).

**Conclusion:**

Results confirmed that pairing of modules to obtain joint kinematics affects the accuracy compared to segment kinematics. Overall, AHRS are a suitable solution for clinical evaluation of biomechanics under the multi-segment tasks performed although the variation in accuracy should be taken into consideration when judging the clinical meaningfulness of the observed changes.

## Background

Inertial measurement unit (IMU) is a term used to describe an integrated sensor package comprised of accelerometers, measuring linear acceleration, and gyroscopes, measuring angular velocity. Combining an IMU with magnetic sensors creates what is known as an attitude and heading reference system (AHRS) where data from all the sensors can be fused together to provide a 3D orientation estimation of the platform with respect to a reference inertial frame based on magnetic North and Gravity. In literature, AHRS are also sometimes referred to as magnetic and inertial measurement unit (MIMU), magnetic angular rate and gravity sensor (MARG) or inertial and magnetic measurement unit (IMMU). Over the past decade, researchers and clinicians have used AHRS to measure segments and joints kinematics in a wide variety of contexts including assessment of age-related kinematic changes, identification of neurodegenerative disease impairments and progression, assessment of rehabilitation evolution, ergonomics evaluations and assessment of sports biomechanics [1–9]. The accuracy of orientation data provided by AHRS has been studied in controlled conditions. These studies demonstrated that velocity, types of motion and environment all affect the quality of the orientation data [10–16]. However, the extent of these effects on human motion remains unclear. To this day, most validation studies concentrate on a single task (mainly levelled gait assessment or handling tasks) performed by a limited number of participants [1–14] and measured on a limited number of segments [13, 17–34]. The methodology used in those studies also varies (difference in systems used, anatomical calibration and referencing…), making it difficult to understand the global scope of AHRS accuracy results in the current literature. For example, Plamondon et al. have shown a significant decrease in trunk angle accuracy with increasing velocity of handling tasks [26]. Interestingly, the impact of velocity on a specific segment accuracy tracked during a definite task is not enough to extrapolate on the effect of velocity on accuracy for another segment or during another task. Among the few studies that have looked at the kinematics accuracy of AHRS in a sit-to-stand context, two studies used a personalized fusion algorithm and optimized gains and cut-off frequencies to get optimal results in angular accuracy [31, 32]. Giansanti et al. justify this approach saying that the frequency domain involved in a sit-to-stand transfer is specific to this task, hence requiring an adaptation of the fusion algorithm [31]. Again, such statement raises concerns about the ability of AHRS to assess mobility equally in varying contexts. Understanding of such possible limits becomes crucial when considering the use of commercially available AHRS to evaluate mobility in different tasks and even more crucial when considering the emerging trend to assess mobility in free-living environments. Errors in assessing accuracy of the movement include: errors associated to the sensors themselves (conditions of motion, magnetic environment, and position of the sensors), soft tissue artefacts associated with the fixation of the modules and errors associated to the anatomical referencing process [34–37]. Recently, Robert-Lachaine et al. have assessed accuracy of multiple segments during a variety of handling tasks [38]. Their study has shown that biomechanical model difference is a major contributor to the error when assessing joint accuracy from a specific inertial system compared to an optoelectronic camera-based system using both their own biomechanical model. Accuracy studies should therefore define the methodology considering these differences and in accordance with the goal pursued [19, 38]. The study from Robert-Lachaine et al. performed on a variety of handling tasks also confirmed that joint accuracy is affected by task duration and complexity, reinforcing the need for in-context accuracy assessment studies. To our knowledge, no study has verified the impact of task complexity on sensor kinematics accuracy for different mobility tasks and none attempted to go one step further, analyzing the impact of the nature of the motion (velocity and type of motion) on accuracy.

The objective of the present study is to evaluate the variation in sensor kinematics accuracy of the orientation data from commercially available AHRS positioned on the head, the upper back, the pelvis, the thigh, the shank and the foot during multiple tasks realized at different paces in order to better understand their optimal use in biomechanics. Specifically, this paper aims at (1) comparing accuracy values in estimating the change in orientation of a segment as well as relative change in orientation of contiguous segments over a variety of mobility tasks (sit-to-stand transition, walking and turning) and across multiple segments; and (2) extrapolating the accuracy results to a broader range of conditions using characteristics of the movements (velocity and type of motion) produced during these tasks.

## Methods

The present study is a concurrent validity study assessing AHRS sensor kinematics accuracy in comparison of a camera-based motion capture system through a variety of tasks (sit-to-stand, walk, turn), measured at multiple segments and joints. The study concentrates on the sensor kinematics aspect of accuracy and therefore does not consider the anatomical referencing process.

### Measurement systems

**Fig. 1:**
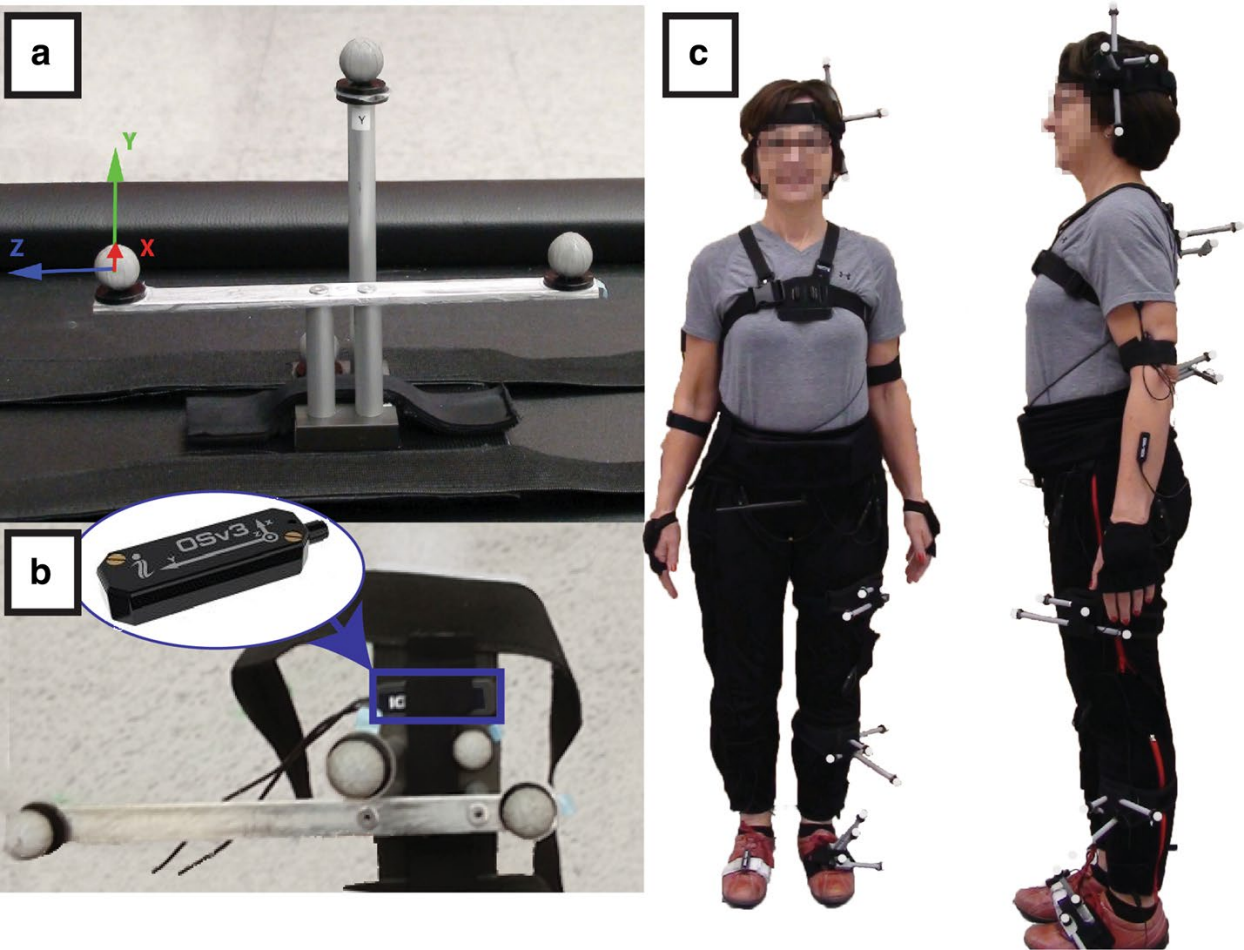
Measurement system and set-up. **a** Rigid bodies comprised of 4–5 passive markers were created to enable the kinematic tracking of the sensors using the Vicon optoelectronic motion-capture system. **b** AHRS were solidly affixed to those rigid bodies using double-sided tape and straps in order to minimize relative motion. **c** The bundles hence created (rigid bodies + AHRS) were positioned on the participants’ dorsal side of the left feet (on the shoe), halfway in the medial surface of the left tibia, two-thirds up the lateral fascia of the left leg, at pelvis level (L5), upper back (about T2) and on the side of the head, above temporal level

The IGS-180 (Synertial) was the selected system to be evaluated in the current study. It is composed of 17 AHRS (model OSv3 also called OS3D, Inertial Labs) enabling full-body kinematics assessment. Its 17 AHRS are wired up into four branches which are, in turn, connected to a mobile processing unit (MPU) worn at the participant’s waist. Specifications details for the AHRS are given in Table 1. The system’s embedded fusion algorithm is a motion-adaptive quaternion-based complementary filter with separate linear acceleration detector, and magnitude disturbance detector. An optimized set of tuning parameters for human motion capture is available from the company and was used for the present study. Data were acquired at 60 Hz and then transmitted to a PC wirelessly. A camera-based motion capture system (Vicon) comprised of 12 cameras (8 MX20, 4 T40) positioned in a capture volume of 3 m × 5 m × 2 m, was used as a gold standard to establish the accuracy of the AHRS. To capture 3D orientation and position of AHRS, rigid bodies made of reflective markers were created and solidly affixed to the AHRS targeted for evaluation (head, upper back, pelvis, thigh, shank and foot). The created bundles were then securely attached to their dedicated limbs using Velcro straps as shown in Fig. 1. Specific care in positioning those bundles was taken in order to minimize soft tissue artefacts, although such issue is not of a direct concern for the present accuracy study as the goals pursued regard sensors kinematics assessment. The two systems are fixed on the same rigid body, which ensures both systems measure the exact same motion. Gold standard data were acquired at a frequency of 100 Hz. Accuracy of the gold standard set-up was verified using the dynamic evaluation process described by Lebel et al. and determined to be 0.002° ± 0.399° for the current protocol [39]. Quality control on the data (visibility and outliers) was performed for each trial to ensure reliability of the gold standard.

**Table 1:** OSv3/OS3D specifications

	Inertial sensors		
	Gyroscopes	Accelerometers	Magnetometers
Range	±1200 °/s	±2 g	±2 Gauss
Resolution	0.07 °/s	0.2 mg	–
Bandwidth	50 Hz	22 Hz	20 Hz
Noise	0:03 °/s√Hz	0.2 mg√Hz	150 µG/√Hz
Bias stability	0.1 °/s (RMS)	1 mg (RMS)	–

	Orientation data	
	Attitude (pitch and roll)	Heading (yaw)
Static accuracy	0.2°	1°
Dynamic accuracy*	1° RMS	<2° RMS
Resolution	0:01°	0:01°

* May vary with the type of motion performed

### Participants

Twenty asymptomatic adults aged between 18 and 83 years old (mean age = 49.9 years old) participated in the study after giving their inform consent following the procedure approved by the CSSS-IUGS ethics committee. Recruitment was targeted to ensure variability in the anthropometrical characteristics (height, weight) and age of the participants so to get variability in the performance and motions across individuals during the tasks. The sample was composed of 50% male and the desired variations in anthropometric characteristics was achieved (height variation 1.50–1.92 m, mean height 1.68 ± 0.11 m; weight variation 49.1–106.8 kg, mean weight 73.4 ± 15.4 kg).

### Experimental protocol

Participants were asked to perform a 5 m timed up and go (TUG) at different paces guided by vocal instructions (i.e. comfortable speed or fast yet safe pace). The TUG is a complex task which combines basic mobility tasks (sit-to-stand, walk and turn) [40]. Although the traditional version of the TUG is 3 m, a 5 m version was used in this study in order to get a better sample of the walk while remaining within the volume of capture of the camera system. The determined TUG path ensured a minimal clearance of 1 m to any external ferromagnetic material (e.g. cabinets, computers, etc.) to be consistent with literature’s recommendations [41]. Prior to each trial, the inertial system was initialized with the participant positioned on a magnetically cleaned spot, standing on a 20 cm high step, in neutral posture (i.e. standing straight, facing forward, palms facing the body). Trials were initiated with the participant sitting down on an armless chair as immobile as possible. Upon signal, the participant would stand-up (STS), walk for 5 m along a predetermined path, turn around and come back to the chair. Each condition (i.e. comfortable and fast speed) was repeated three times. Trials were then manually segmented in the temporal space using Nexus version 1.8.2, the data acquisition and analysis software for the Vicon System, with the following guidelines:

(i) STS: Starts at motion initiation (any segment) and ends when the head reaches its maximum vertical displacement.
(ii) Walk1: Starts when STS ends and goes on until alignment head/trunk/foot is modified.
(iii) Turn: Starts when alignment head/trunk/foot is modified and ends up when this same alignment is re-established.
(iv) Walk2: Starts when the turn ends and goes on until the alignment head/trunk/foot is again perturbed.
(v) Turn and Sit: Starts when alignment head/trunk/foot is modified and ends up when the person is sitting down on the chair.

AHRS data were resampled to match the optical gold standard frequency and synchronized in post-processing using a cross-correlation approach. Data processing was performed using Matlab (R2015a from Mathworks) while statistical analyses were performed using SPSS (v23.0.0 from IBM).

### Data reduction

The current study concentrates on absolute and relative sensor kinematics accuracy assessment in a biomechanical context. In other words, the methodology allows us to isolate orientation estimation errors due to the sensors, enabling a better understanding of the capabilities of such systems for biomechanical evaluation. As such, accuracy was established using the angular deviation of the AHRS *segment* orientations to the reference *segment* orientations, measured by the optical gold standard [42]. Specifically, the orientation of each *segment* and *joint* was expressed in terms of the orientation at the beginning of the targeted task and the angular deviation was calculated from the quaternion distance, as represented in 1.

$$\underline{q}_{AHRS} = \left[ {\begin{array}{*{20}c} {{ \cos }\left(\frac{{\theta_{AHRS} }}{2}\right)} \\ {a_{AHRSx} sin\left(\frac{{\theta_{AHRS} }}{2}\right)} \\ {\begin{array}{*{20}c} {a_{AHRSy} sin\left(\frac{{\theta_{AHRS} }}{2}\right)} \\ {a_{AHRSz} sin\left(\frac{{\theta_{AHRS} }}{2}\right)} \\ \end{array} } \\ \end{array} } \right] = \left[ {\begin{array}{*{20}c} {\eta_{AHRS} } \\ {\varepsilon_{AHRS} } \\ \end{array} } \right] \,{\text{as the change in orientation of the AHRS}};$$

$$\underline{q}_{opto} = \left[ { \begin{array}{*{20}c} {{ \cos }\left(\frac{{\theta_{opto} }}{2}\right)} \\ {a_{optox} sin\left(\frac{{\theta_{opto} }}{2}\right)} \\ {\begin{array}{*{20}c} {a_{optoy} sin\left(\frac{{\theta_{opto} }}{2}\right)} \\ {a_{optoz} sin\left(\frac{{\theta_{opto} }}{2}\right)} \\ \end{array} } \\ \end{array} } \right] = \left[ {\begin{array}{*{20}c} {\eta_{opto} } \\ {\varepsilon_{opto} } \\ \end{array} } \right]{\text{ as the change in orientation of the rigid body}};$$

1 $$\theta_{diff} = 2{\text{acos}}\left( {\underline{q}_{AHRSal} \cdot \underline{q}_{opto} } \right)_{\omega } ,$$

where $( \cdot )_{\omega }$ corresponds to the scalar part of the quaternion.

Although this approach does not allow to associate the movement with a specific plane of motion (i.e. anatomical reference), global motion characterization has the advantage of focusing on the sensor kinematic accuracy assessment, with minimal consideration for errors due to alignment protocols and/or biomechanical model [39]. Accuracy parameters were then derived from θ_diff_, the orientation difference between the global motion measured by the AHRS and the global motion measured by the optical gold standard.

#### Accuracy parameters

The system’s accuracy was characterized using a number of parameters. First, the root-mean-squared difference (RMSD) was computed for each trial and the mean and standard deviation over the 120 trials (20 participants, 6 trials/participants) are herein reported. The interpretation guidelines listed below for these parameters were used throughout the study to characterize the accuracy. These guidelines were extrapolated from those proposed by McGinley et al. [43] and are:

- RMSD < 2°: good accuracy, within natural variation of an individual’s kinematic parameters;
- 2° < RMSD ≤ 5°: acceptable accuracy;
- 5° < RMSD ≤ 10°: tolerable accuracy, requires consideration in the interpretation;
- RMSD >10°: unbearable accuracy.

Mean peak error (Err_peak_) is also reported to get a better portrait of the possible errors one might face while using AHRS to evaluate a kinematic parameter at a specific point in time. To facilitate comparison with published literature, mean absolute differences (MAD) are also reported. Reliability of the system is assessed with Ferrari’s version of the coefficient of multiple correlation (CMC) [44]. This specific version of the CMC allows for similarity assessment between waveforms, ignoring inter-cycle variability, but taking into account the effects of offset, correlation and gain. The guidelines proposed by Ferrari et al. were used to interpret the agreement between the curves [19]:

- 0.65–0.75: moderate agreement.
- 0.75–0.84: good agreement.
- 0.85–0.94: very good agreement.
- 0.95–1.00: excellent agreement.

The described accuracy assessment (RMSD, Err_peak_, MAD and CMC) was performed for the sensors located on the targeted segments (head, upper back, pelvis, thigh, shank and foot) and joints (hip, knee, ankle and trunk or upper back relative to pelvis) for the sit-to-stand transition, the walking task and the turn.

#### Effect of velocity and type of motion

**Fig. 2:**
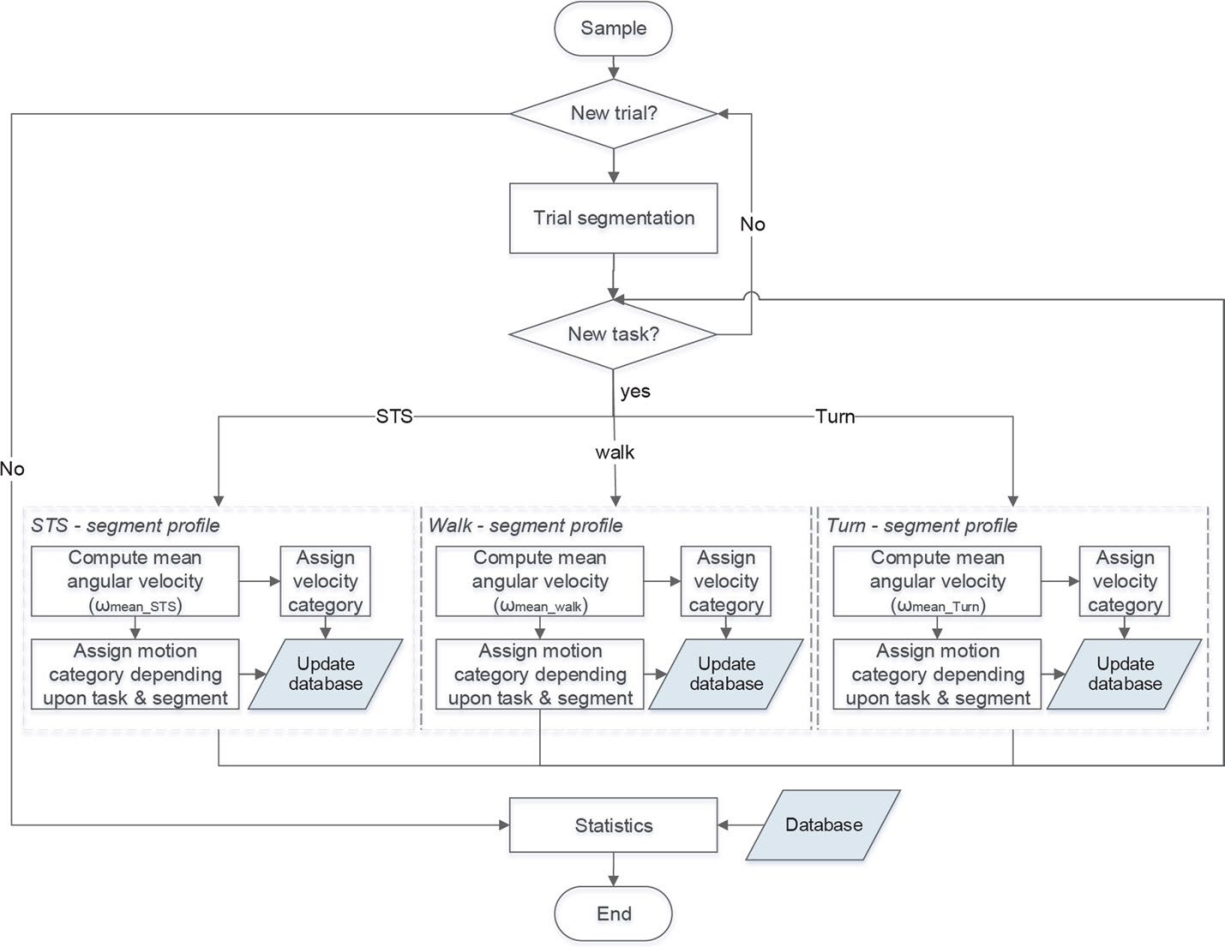
AHRS data segmentation and analysis. Each trial was segmented into basic tasks (sit-to-stand, walk and turn). For each data segment, mean angular velocity was calculated and assigned a velocity category. Based on the data characteristics, a type of motion was also determined for the data segment. The information on velocity category and type of motion for each specific data segment is then stored into a database for further analysis upon completion of the trials classification process

As discussed earlier, many authors have shown, in controlled conditions and for human motion, that an increase in velocity worsens the orientation data accuracy [11, 13, 14, 26], but current evidence is insufficient in determining the extent of the velocity effect on a variety of tasks measured at different segments. The proposed approach aims at identifying the optimal range of operation of AHRS in a regular biomechanical context. This approach not only enhances the comprehension of the system behaviour, but it is also meant to provide insights regarding the system accuracy behaviour for movements other than the ones specifically tested in the protocol or for an impaired population. The first step towards this goal is to characterize each part of the trials according to specific criteria regarding velocity and types of motion as shown in Fig. 2. Velocity categories were created in order to cover the full range of tasks’ mean angular velocities observed over the total 120 trials (20 persons, 6 trials/person, 4 task samples/trial, 6 segments tracked/trial or 4 joints tracked/trial = 2880 data for *segment* tracking accuracy and 1920 data for *joint* tracking accuracy). Size of the bins was fixed and determined with the lowest 30% cut-off point. Following this process, six categories were created (cat1 <25°/s; cat2 25°–50°/s; cat3 50°–75°/s; cat4 75°–100°/s; cat5 100°–125°/s; cat6 ≥125°/s). Representativeness of each category within the data sample was verified. Following normalization of the data, a Welch ANOVA was performed to determine if the effect is statistically significant or not, followed by Games-Howell post hoc testing to identify the specific differences if appropriate.

The type of motion was determined based on the predominant motion of the specific segment/joint during the particular task. The possible types of motion identified as most common in daily living activities are:

(i) Quasi-static: Segment is immobile or close to it during the task (e.g. foot during STS);
(ii) Lateral displacement (LateralD): Almost no angular motion is perceived but the segment is moving linearly (e.g. upper trunk during the walking task);
(iii) Single angle change (single angle): Segment performs a single continuous angular motion and then remains in that orientation (e.g. upper trunk during turn);
(iv) Pendulum: Segment moves back and forth during the task (e.g. thigh during the walking task).

Again, Welch ANOVA was performed on normalized data to assess the impact of the type of motion followed by Games-Howell post hoc analysis when applicable.

## Results

Mean absolute sensor kinematic accuracy (i.e. *segment* level) evaluated with RMSD varied between 1.1° and 5.5° depending on the task performed and the module considered, as shown in Table 2. For the same trials, mean relative sensor kinematic accuracy (*joint* level), again evaluated using RMSD, varied between 1.6° and 13.6°. Assessment of similarity between the angular curves produced by the two systems reports a good to excellent similarity for all tasks at a *segment* level with mean CMCs varying between 0.783 and 1.0. However, CMC decreased when evaluated at *joint* level, remaining above 0.85 (i.e. very good agreement) at hip and knee levels throughout the tasks.

**Table 2:** Absolute and relative sensors kinematics accuracy assessed on multiple segments and Joints during different tasks

STS (n = 120)			Walk (n = 240)			Turn (n = 120)		
RMSD	Err_peak_	CMC	RMSD	Err_peak_	CMC	RMSD	Err_peak_	CMC
Absolute sensor kinematics								
Head								
4.0° [2.3°–6.8°]	8.0° [5.3°–13.6°]	0.900 [0.740–0.966]	3.7° [2.2°–5.5°]	8.9° [5.6°–12.7°]	0.819 [0.778–0.879]	4.4° [2.9°–5.7°]	6.8° [5.0°–9.2°]	0.998 [0.998–0.999]
Upper Trunk								
1.5° [1.1°–2.1°]	3.7° [2.5°–4.9°]	0.996 [0.992–0.997]	2.9° [1.8°–4.4°]	6.8° [4.5°–9.6°]	0.853 [0.779–0.897]	3.1° [1.6°–4.7°]	5.6° [3.4°–8.9°]	0.999 [0.998–1.000]
Pelvis								
1.0° [0.8 ° –1.5°]	2.9° [2.1°–4.1°]	0.997 [0.988–0.999]	2.2° [1.4°–3.6°]	5.1° [3.6°–8.4°]	0.842 [0.646–0.946]	1.7° [1.4°–2.9°]	4.0° [3.0°–6.4°]	1.000 [0.998–1.000]
Thigh								
0.9° [0.7°–1.4°]	2.4° [1.8°–3.2°]	0.999 [0.997–1.0]	1.6° [1.1°–3.0°]	4.4° [3.2°–7.7°]	0.981 [0.918–0.991]	1.3° [0.8°–1.9°]	3.3° [2.4°–4.9°]	1.000 [1.000–1.000]
Shank								
1.3° [0.9°–1.7°]	3.2° [2.5°–4.4°]	0.983 [0.974–0.989]	2.4° [1.7°–3.2°]	5.6° [4.0°–7.7°]	0.990 [0.987–0.994]	2.4° [1.7°–2.9°]	4.9° [3.8°–6.6°]	1.000 [0.999–1.000]
Foot								
1.1° [0.6°–2.3°]	2.9° [1.6°–5.7°]	0.935 [0.629–0.986]	2.1° [1.4°–3.2°]	5.7° [4.0°–8.6°]	0.996 [0.967–0.997]	1.7° [1.1°–2.8°]	4.5° [3.2°–8.3°]	1.000 [0.999–1.000]
Global								
1.3° [0.9°–2.2°]	3.3° [2.2°–5.5°]	–	2.4° [1.5°–3.9°]	5.8° [3.9°–9.2°]	–	2.2° [1.4°–3.8°]	4.8° [3.3°–7.5°]	–
Relative sensor kinematics								
Trunk								
2.8° [1.9°–4.4°]	6.4° [4.1°–9.7°]	0.808 [0.744–0.891]	3.5° [2.3°–5.6°]	8.5° [6.1°–12.9°]	0.501 [0.343–0.741]	4.1° [2.6°–6.2°]	8.4° [6.4°–11.8°]	0.598 [0.470–0.663]
Hip								
1.5° [1.1°–2.2°]	3.7° [2.9°–5.0°]	0.997 [0.992–0.998]	2.7° [1.8°–4.4°]	7.1° [5.1°–10.2°]	0.951 [0.910–0.975]	3.4° [2.4°–5.4°]	8.3° [6.0°–11.8°]	0.926 [0.861–0.954]
Knee								
1.4° [1.0°–2.0°]	3.6° [2.8°–4.7°]	0.999 [0.998–0.999]	3.3° [2.5°–4.5°]	8.4° [6.3°–11.2°]	0.982 [0.974–0.988]	3.9° [2.3°–5.4°]	8.1° [5.7°–12.1°]	0.981 [0.965–0.986]
Ankle								
2.5° [1.5°–4.0°]	6.3° [4.1°–10.6°]	0.881 [0.623–0.928]	7.8° [5.7°–11.3°]	20.6° [15.3°–30.4°]	0.712 [0.643–0.772]	9.4° [6.5°–16.6°]	23.5° [16.7°–37.0°]	0.517 [0.219–0.678]
Global								
1.9° [1.3°–3.1°]	4.5° [3.3°–7.4°]	–	3.9° [2.5°–6.7°]	9.7° [6.4°–16.5°]	–	4.6° [2.8°–7.7°]	10.4° [6.7°–18.6°]	–

*RMSD* root mean squared difference, median [IQR]; *Err*_*peak*_ peak error, median [IQR]; *CMC* coefficient of multiple correlation, median [IQR]

### Effect of the type of motion

**Fig. 3:**
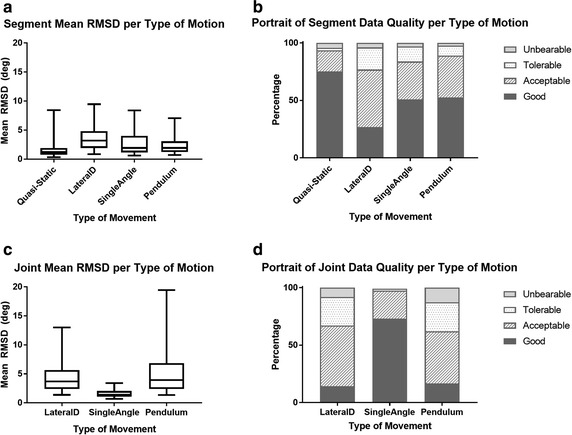
AHRS accuracy per type of motion. RMSD and qualitative classification of trials’ accuracy per type of motion (Quasi-Static, lateral displacement, single angle change and pendulum) for **a**, **b** absolute sensor kinematics (i.e. segments) and **c**, **d** relative sensors kinematics (i.e. joints)

The mean RMSD per category of motion for (A) single sensors (or *segments*) and (C) relative sensors (or *joints*) are shown in Fig. 3. A Welch ANOVA ran on normalized data have shown a statistically significant difference between the categories of motion both at the segment and the joint levels (p < 0.001). At the *segment* level, Games-Howell post hoc analyses revealed that single angle change and pendulum categories both have significantly better accuracy than the movement involving mainly lateral displacement as well as quasi-static conditions (p < 0.001 for both). At a *joint* level, single angle movements were shown to perform significantly better than the other two categories (p < 0.001). The impact of the difference is clearly demonstrated in panels B and D of Fig. 3 which shows the proportion of good (≤2°), acceptable (2°–5°), tolerable (5°–10°) and unbearable (>10°) trials for each category at *segments* and *joints* levels.

### Effect of velocity

**Fig. 4:**
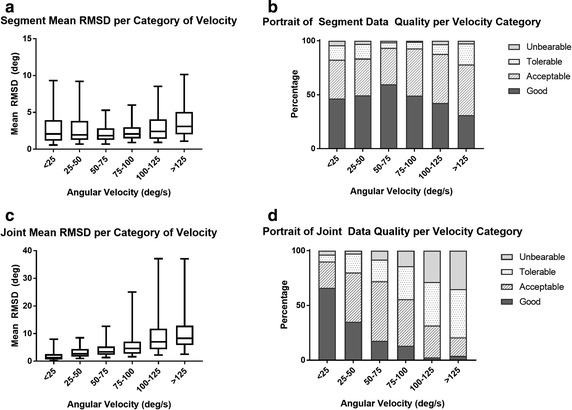
AHRS accuracy per angular velocity category. RMSD and qualitative classification of trials’ performance per category of angular velocity for **a**, **b** absolute sensor kinematics (i.e. segments) and **c**, **d** relative sensors kinematics (i.e. joints)

Effect of velocity is shown in Fig. 4 for all types of motions analyzed together at (A) *segment* level and (C) *joint* level. Welch ANOVA on normalized data revealed a statistically significant impact of velocity in both cases (p < 0.001). Results from post hoc analyses revealed an optimal zone of operations between 50 and 75°/s at *segment* level while this optimal zone is a bit slower at *joint* level, with the 25–50°/s category being identified as optimal. Again, panels B and D illustrate the proportion of good (≤2°), acceptable (2°–5°), tolerable (5°–10°) and unbearable (>10°) trials for each category at *segment* and *joint* levels.

## Discussion

The current study first aimed at providing a portrait of AHRS sensor kinematics accuracy over a variety of tasks measured with sensors positioned at various segments and using different approaches (absolute sensor kinematics or segment vs relative sensor kinematics or joint). As such, mean root-mean-squared sensors accuracy ($\overline{RMSD}$) was shown to vary between 1.1° and 5.5° depending on the task performed and the segment considered, while mean relative sensors accuracy was reported to be between 1.6° and 13.6° over the same trials. $\overline{RMSD}$ varied between tasks and segments/joints, tending to prove that AHRS algorithms are better adapted to certain conditions. However, $\overline{RMSD}$ remained acceptable for all segments throughout the tasks (STS, walk, turn) except for the head during STS based on the guidelines suggested by McGinley et al. for acceptability of kinematics measurement [43]. Using the same 5° guideline on relative sensor accuracy, the tracking of the hip and the knee were both shown to be acceptable throughout the three tasks as well as the trunk during the sit-to-stand transition and the walk. The good reliability reported per task and segment/joint tends to prove that the system is quite robust to small variations in the conditions of motion (e.g. distance of the sensor to the joint).

Using a different type of AHRS and a biomechanical model decomposing the joint angle into anatomical referenced angles, Zhang et al. had identified mean errors within the same range for the hip, slightly higher for the knee and clearly lower for the ankle when computed over a single gait cycle (Hip: 2.5° F/E, 4.8° A/A, 3.0° I/E; Knee: 1.9° F/E, 5.1° A/A, 2.7° I/E; Ankle: 2.2° F/E, 1.8° A/A, 1.8° I/E) [23]. Ferrari et al. have reported comparable CMCs for the hip and the knee but higher CMCs for the trunk and the ankle for levelled walking at self-selected speed [19]. The differences observed could be in part explained by the bigger variation in the conditions of motion introduced in the current study (20 participants aged between 18 and 83 years old walking at slow and fast paces compared to four participants aged between 26 and 31 years old, walking at self-selected pace) which produces a larger variation in the angular velocity spectrum of the motion as well as in the linear acceleration associated with the motion. As far as the STS is concerned, the RMSD and CMC values obtained for the upper trunk and the pelvis using the system’s tuning parameters recommended for human motions are within the same range as the values reported in the literature, although those studies argued that there is a need for specific tuning of the fusion algorithm to obtain these performances [31, 32]. To the authors’ knowledge, no validation study has addressed the accuracy of the orientation data during the turning phase. However, a recent publication has shown that turning performance is compromised in diseases such as PD [45]. Yet, spatiotemporal characteristics of turn have shown weak reliability when tested during five consecutive days on twelve community dwellers older adults [46]. Considering the low reliability of current turn characteristics combined with the fact that turns may occur far more often than straight walking during normal in-home activity, it makes sense to deploy the required efforts to derive specific parameters for that task. The level of error obtained for segments tracking suggests that such orientation could be useful to enhance the analysis of the “quality” of a turn.

The accuracy portrait presented in this paper also supports the idea that pairing AHRS to get joint angular motion does affect the accuracy [12, 14, 15]. Indeed, global mean absolute sensor accuracy varied between 2.6° and 3.2° depending on the task performed, while it varied between 3.2° and 7.2° for relative sensor kinematics accuracy over the same tasks. Furthermore, a diminution in the agreement between the systems was shown when considering joint angle variations versus segment orientation variations (0.82 ≤ CMC ≤ 1.0 at segment level, 0:46 ≤ CMC ≤ 0.998), again supporting the idea that pairing of modules affects accuracy. The extent of the differences tends to show that there is more to it than only measurement error addition. The difference in velocity between the segments as well as the difference in environment may also contribute to this error. Hence, when information of interest can be measured using a single module versus two (e.g. trunk motion during sit-to-stand which can be assessed looking at the change in orientation of the upper trunk module or change in relative orientation of the upper trunk with respect to the pelvis), a single module approach should be prioritized as it seems more robust.

The current study also aimed at extrapolating the accuracy results to better understand the variations based on the movements characteristics and, in the meantime, identifying an optimal region of operations for commercially available AHRS in clinical biomechanics. As such, it was shown that the variation in accuracy level is partly related to the type of motion performed as well as to the level of angular velocity registered by the gyroscope. Crossing those analyses together, however, revealed that the velocity of so-called “single angle motion” remained below 50°/s in the vast majority of cases which may explain its superiority to global pendulum motion which is present throughout the range of velocity categories. Hence, angular velocity seems to have a predominant effect on accuracy, with an optimal region of operations identified for the IGS-180 between 25°/s and 75°/s. As a guideline for interpretation, it can be noted that 85% of the walking tasks were classified between 25°/s and 75°/s with regards to the hip, 80% were identified in categories between 50°/s and 100°/s at the knee and 85% of the ankle trials were classified between 50°/s and 125°/s. In quasi-static conditions (i.e. velocity <25°/s), the magnetic environment is also quasi-constant and may be perturbed. If the module remains in these conditions for longer than a system-specific period of time, the magnetic compensation algorithm will identify this new environment as the goal reference and slowly adapt its global reference to meet this new environment, causing a drift in the data. In the current study conditions, this situation happened mainly at foot level during the STS, explaining the variability shown in the results. Indeed, common building construction material perturbed the magnetic field at the floor level, which perturbations decreased in importance as we move away from the floor [41, 47]. The sensors being static for a certain period of time prior to initiating the STS, the drift was, in some cases, present. For researchers and clinicians, this translates into a required increased awareness of environmental conditions when AHRS are used to assess quasi-static motions (e.g. balance tests). At the other end, would a researcher want to assess the kinematics of figure skating, he should be aware that orientation accuracy may worsen during specific spins. Hence, depending on the goal, it may be required to fine-tune the fusion algorithm to obtain reliable results. Overall, the variation in accuracy portrayed in this study demonstrate the importance of knowing the accuracy of the measurement system for the specific context of use. As such, the analysis of the impact of the type of motion and the velocity on accuracy allows to generalize the conclusions for different types of motion. Furthermore, such conclusions may be used to develop automatic quality control on data to increase reliability, as proposed by Lebel et al. [48].

The main limitation of the study is the fact that the analysis was performed on a single commercially available system, somehow limiting its generalizability. However, comparative studies in controlled conditions have shown that velocity and types of motion impacts are common to some of the most popular commercial systems (Xsens MTx, Opal APDM and Inertial Labs OSv3) [14, 15]. Xsens also clearly states in their MTx specifications datasheets that the orientation dynamic accuracy may vary depending on the type of motion performed [49]. It is therefore presumed that current commercially available AHRS for biomechanics will have similar behaviour in terms of optimal region of operations and that researchers should be aware of that.

The other important limitation of this study concerns the fact that it concentrates on the evaluation to the sensors kinematics without consideration of the biomechanical model. This specific methodological choice was made in order to enhance the understanding of the technological limitations themselves. Clinicians and researchers should, however, be aware that the accuracy of any technological equipment used for mobility assessment does not only depend upon the technological capability to measure accurately motion in the context of use, but also on soft tissue artifacts due to the positioning and fixation of the modules as well as errors due to anatomical calibration and referencing [22, 35, 36, 38, 50, 51]. To the authors’ opinion, these issues should be addressed separately as they are of a different nature.

## Conclusions

The results from the present study emphasize the possibility of using AHRS for clinical evaluation of biomechanics, but the accuracy of kinematics data varies according to the task performed and the segment/joint tracked. Pairing of modules to assess joint kinematics also affects accuracy compared to segment kinematics. The observed variation in accuracy was partially explained by the varying angular velocity of the segments and the environment in which the movements are performed. This needs to be taken into consideration by clinicians when judging the clinical meaningfulness and limitations of the observed changes in joint kinematics captured by AHRS.

## Abbreviations

AHRS: attitude and heading reference system
CMC: coefficient of multiple correlation
IMU: inertial measurement unit
MAD: mean absolute difference
MPU: mobile processing unit
RMSD: root-mean-squared difference
STS: sit-to-stand transfer
TUG: timed-up and go
